# Impact of a Primary School Health Promotion Programme on Adolescents’ Health Behaviour and Well-Being

**DOI:** 10.3390/children11080919

**Published:** 2024-07-30

**Authors:** Gabriella Nagy-Pénzes, Ferenc Vincze, Ágnes Víghné Arany, Éva Bíró

**Affiliations:** 1 Department of Public Health and Epidemiology, Faculty of Medicine, University of Debrecen, 4032 Debrecen, Hungary; vincze.ferenc@med.unideb.hu (F.V.); biro.eva@med.unideb.hu (É.B.); 2 Independent Researcher, 4028 Debrecen, Hungary; egtan01@gmail.com

**Keywords:** school health promotion, primary school, health behaviour, well-being, intervention, adolescents

## Abstract

Background/Objectives: Schools can play a key role in promoting health among adolescents, and Hungarian legislation gives them sufficient space to do so. In our study, we examined the impact of a multiyear school health promotion programme on pupils’ health behaviour and well-being. Methods: We carried out our investigation in an intervention and a control primary school in Hungary. All 5th and 7th grade pupils were invited to participate in the questionnaire-based survey between 2017 and 2021. The effect of the intervention was quantified using univariate and multivariate logistic regression analyses. Results: Our results show that for those behaviours where pupils’ personal choices had a greater influence (unhealthy eating, smoking, screen time), the health promotion programme was more effective. For those behaviours where family background and parental influence were more pronounced (healthy eating, physical activity), the intervention had less impact. Self-perceived health was better in the 7th-grade intervention group. Conclusions: Our findings are in line with the conclusion of systematic reviews that more intensive, longer-term, multi-behavioural school health promotion programmes can be effective in promoting positive behaviour. To be more effective, it would be worth using a well-structured curriculum, well-developed teaching materials, and greater involvement of teachers, parents, and various local organisations.

## 1. Introduction

For people to take control of their health and would like to maintain and improve it, it is vital that they see health as a value. This process could be enhanced during individual socialization if health promotion is a part of it. The family and school, as the main socialization agents of the individual, play a major role in the process of value formation, as social and sociocultural values are transmitted in some form. The family, as the primary socialization agent, plays a significant role in the development of the health and health behaviour of the younger generation by passing on its values and setting an example. Furthermore, if health is one of the school’s values, it can also do a lot for the health of the growing society through its system of norms and education [1,2]. In addition, a high sense of belonging at school has a positive impact on the psychological health of adolescents [3], and both are important from the point of view of school attendance and engagement in health promotion programmes.

Adolescence is the stage of a person’s life that marks the beginning of adulthood—biologically, mentally, and spiritually [4]. The World Health Organization (WHO) defines this period as aged between 10 and 19 [5]. The bio-socio-psychological transition indicates the end of childhood so in addition to biological, social, and psychological changes, new and essential forms of behaviour emerge [4,6]. The health-conscious behaviour of children and adolescents is not usually developed individually but rather through a comprehensive socialization process, as explained above. During the first years of life, children’s health behaviours are significantly influenced by their parents. As children enter adolescence and the socialization space expands, environmental influences (such as peers and friends) and the role of individual choice come to the fore in determining their health behaviour. Consequently, many health-related attitudes and behaviours are attempted, acquired, or discarded during this phase of life. The eating habits and physical activity of adolescents change with age, and this is the period of contact with tobacco products, alcohol, or other psychoactive substances [7,8,9]. The vast majority of behaviours and habits acquired in adolescence persist into adulthood [10], so these behaviours not only influence adolescent health but also could determine adult health.

The results of recent surveys show that Hungarian adolescents have more unhealthy diets and worse tobacco and alcohol consumption habits than the international average. They are also more likely to be overweight, less likely to rate their health as excellent, and have higher rates of psychosomatic symptoms [8,11,12,13]. Based on the results of these surveys, there is a particular need for effective health promotion activities among Hungarian adolescents.

Educating young people about a healthy lifestyle can be easily implemented in schools. Health promotion activities tailored to the target group during adolescence can more effectively influence their lifestyle and thus their health status in the long term [14]. Health education can be a good tool for developing health literacy and converting acquired knowledge into favourable behaviour [15]. The characteristics of evidence-based, effective school health promotion programmes can be described as focusing on both academic and social outcomes; being comprehensive and holistic; linking the school to other sectors, including health organisations; being sufficiently deep and long term; having a significant impact on students’ academic and social development [16].

Several review studies published in recent years have shown that health promotion activities in schools can be effective in fostering positive health behaviours in students, depending on the method and intensity. Although their impact is not very large, they can be important at the population level [17,18,19,20,21]. The American Centers for Disease Control and Prevention (CDC), the WHO, and the School for Health in Europe Network Foundation (SHE) of which Hungary is also a member, all recognize the importance of a comprehensive approach to health promotion in schools. Moreover, all three organisations make recommendations for health promotion in schools [22,23,24,25,26,27].

In Hungary, several laws and regulations address the protection and promotion of the health of children and adolescents [28,29,30,31,32,33,34,35]. They cover, among others, daily physical education and stipulate that every educational institution must implement holistic health promotion (HHP) [34]. The HHP is a universal, whole-school approach where health promotion should be part of the everyday life of the school. The HHP includes four health promotion tasks in the daily work of schools, namely, healthy eating, physical activity, pedagogic methods to promote mental health, and improving the health literacy of the children, involving the entire school, parents, and the environment of the school [36]. In addition, health education is one of the objectives of the National Core Curriculum (NCC), which introduces students to healthy lifestyles as a part of their subjects [35].

However, relatively little is known about how successful schools are able to fulfil all of these criteria, which is why we can relate to the results of individual school health promotion programmes if we are interested in the effectiveness of the different activities. An example of this kind of programme is presented below.

### 1.1. A Holistic Long-Term Hungarian School Health Promotion Programme

A health promotion programme entitled “Developing Healthy Lifestyle in the Eco-School” was launched in the 2008/2009 school year in a Hungarian primary school as part of the Eco-School Programme. (The Eco-School title can be awarded to educational institutions that deal with environmental awareness, education for sustainability, and environmental and health education in a well-thought-out, systematic, and everyday practice within an institutional framework, and the work of the school is also linked to the local community [37,38,39].) The programme was coordinated by the school management, the teaching staff, and the local public health institute under the professional guidance and mentoring of a health education curriculum expert.

The programme introduced health promotion as a subject in one class of each of the eight grades, in the second half of the school year, for one hour per week, with 18 lessons per study year integrated into the local curriculum. The aim of the programme was to improve the physical, mental, and social health of the pupils by improving their health knowledge and related skills and abilities and by shaping healthy attitudes and behaviour [40,41]. The programme combines environmental education with health promotion.

The number of students participating in the programme increased in an ascending system from the second year, so that from the 2016/2017 school year, all students at the school participated in the health promotion programme. From the 2012/2013 school year, a new National Core Curriculum [35] put more emphasis on health education and promotion, so the existing health promotion programme was adapted accordingly, and health education was then integrated into different subjects (e.g., homeroom, science subjects), linked to the relevant curricular content [40].

The programme consisted of three main parts: (1) special training in health promotion for teachers, (2) health promotion lessons with a well-structured curriculum based on authentic workbooks (educational tools and materials) for pupils, and (3) other health promotion activities through school and city programmes involving parents and external experts.

#### 1.1.1. Teacher Training in Health Promotion

At the beginning of the programme, health promotion was preceded by in-service training for teachers at the school, delivered by a health education curriculum expert as external professional support. The 10 h training course prepared teachers to provide health promotion activities by shaping their preventive and ecological approach. Following the practical training, a health promotion demonstration lesson was organized in all eight grades, which also served as a methodological guide. Each demonstration lesson was followed by a problem-focused professional consultation with the schools’ management and external professional partners (health education curriculum experts and public health and health promotion specialists). The theoretical and practical training has prepared the teachers involved in the programme for more effective health promotion activities. At the beginning of each school year, teachers participating in the programme received in-service training and were also given demonstration lessons, which teachers from other schools were able to attend [40].

#### 1.1.2. Educational Tools and Materials Used for Health Promotion

The health promotion lessons followed a structured curriculum based on a series of health promotion workbooks for grades 1 to 8. The workbooks were edited and written by a health education curriculum expert and a teacher committed to health promotion. The authors’ professional experience has contributed to the quality and didactic content of the workbooks [42].

Workbooks contain theoretical and practical knowledge and exercises that can be used in everyday life, and attitude-building content also encourages pupils to develop health-conscious attitudes and behaviours. The aim of the workbooks is to support the healthy physical, mental, and social growth of pupils, taking into account the age specificity of each grade.

The topics of the workbooks are built on each other from grades 1 to 8, expanding the curriculum on healthy lifestyles, self-knowledge, and social health according to age specificity. Keeping the above in mind, the workbooks are based on three modules: (1) healthy lifestyle, (2) family and social life, and (3) development of self-knowledge. The content of each module is presented in Table 1.

The modules are related to each other, and each topic is covered from several aspects, but they are consistent with the curriculum as a whole. Systematic tasks and exercises aim to shape a holistic view of how the physical and mental health of a person is related to their social, built, and natural environment.

The modules of the health promotion workbooks were accompanied by final knowledge tests (knowledge tests I, II, and III) to assess the knowledge acquired by the pupils after each module. In the Healthy Lifestyles programme, these tests were used to measure changes in pupils’ knowledge (pupils completed the test before and after each module), and the results were used to determine whether pupils had acquired the appropriate knowledge during the module. Between 2008 and 2016, tests carried out by schools and health education curriculum experts concluded that pupils’ knowledge of healthy lifestyles improved successfully [40].

#### 1.1.3. Other Health Promotion Activities through School and City Programmes Involving Parents and External Experts

In addition to the health promotion lessons, pupils were also able to participate in other health promotion and health education activities. In the school’s pedagogical programme, health promotion goes beyond the classroom and is part of the day-round education, in the form of art activities, sports clubs, talent programmes, various action programmes, projects, and theme days and weeks. Pupils had the opportunity to participate in programmes organized by external experts (e.g., public health bodies) and other organisations associated with them, which contributed to the pupils’ environmental and health education (e.g., training in first aid). Pupils were regularly involved in the maintenance of the schoolyard (e.g., planting shrubs and flowers), learning about herbs in herb garden care, and composting. All of this brought them closer to the joy of gardening and the respect and love of nature.

The school has also paid attention to how parents can help implement health and environmental education programmes. Parents with expertise in specific subjects were involved in the organisation and implementation of school programmes.

The school physician and the school nurse carried out age-related examinations of the pupils, administered compulsory vaccinations, and contributed to prevention-related tasks (e.g., lectures on healthy lifestyles and showing educational films) in coordination with the school management.

#### 1.1.4. Methods of “Developing Healthy Lifestyle in the Eco-School” Health Promotion Programme in Light of the COM-B Model

The COM-B model provides a well-utilized theoretical framework for the planning of effective interventions inducing behaviour change. The model was developed by a group of psychologists (Michie S. et al.) by synthesizing all known theories describing behavioural changes. According to the COM-B model, three key factors influence an individual’s behaviour: capability (the physical and psychological ability to perform a behaviour), opportunity (the physical and social environment that enables the behaviour), and motivation (the reflective and automatic mechanism that activates/inhibits the behaviour); furthermore, ability and opportunity independently affect motivation [43].

The COM-B model was created after the start of the school health promotion programme detailed above, but many elements of the programme (structure of the curriculum, shaping abilities, skills, habits, attitudes, creating a supportive environment) can be organized accordingly (Appendix A). Therefore, it can be said that the programme in its conceptual form can be capable of creating a more favourable health status and health behaviour.

The aim of our study was to examine the impact of the above-described multiyear school health promotion programme on pupils’ health behaviour and well-being.

## 2. Materials and Methods

### 2.1. Study Population

We carried out our investigation in two primary schools in Debrecen, the second largest Hungarian city located in East Hungary, between 2017 and 2021. One of them was the intervention (participating in the multiyear school health promotion programme described in the introduction), and the other was the control school. They did not differ significantly in terms of age or sex, each was located in a similar socioeconomic part of the town, they had similar class sizes and class numbers, and both institutes had the same educational programmes.

We invited all 5th and 7th grade pupils to participate in the survey. We sent consent forms to the parents of all pupils; only pupils who provided both parental consent and active consent themselves were eligible to participate. The study was conducted according to the guidelines of the Declaration of Helsinki and approved by Hungary’s Medical Research Council Scientific and Research Committee (49460–5/2016/EKU).

### 2.2. Characteristics of the Control School

To ensure comparability when selecting the control school, we ensured that it had fundamentally similar characteristics as the intervention school, so it is also based in Debrecen and is socioculturally similar to the intervention school. In 2016, this school was declared an Eco-School, so it is also important for them to develop environmentally conscious lifestyles.

The control school did not have a health promotion programme similar to the intervention school, i.e., no specific subject (before HHP and NCC 2012) and curriculum based on thematic health promotion workbooks was used, and teachers were not specifically trained to implement health promotion activities in the school. However, the school’s pedagogical programme includes a compulsory health promotion programme, compulsory daily physical education and health education integrated into the subject areas in accordance with Hungarian laws [28,29,30,31,32,33,34,35].

The school organizes extracurricular health promotion activities in the form of theme weeks and days, annual game competitions and sports clubs. The focus of these activities is mostly physical activity, and they are also involved in several city and international sporting events. The school doctor and the school nurse carried out age-related health checks among the pupils, administered compulsory vaccinations and participated in other prevention activities (e.g., lectures on healthy lifestyles) in coordination with the school management.

The school participates in city programmes when possible and cooperates with other health organisations (e.g., since the 2009–2010 school year, they are partners of the Hungarian Red Cross; since then, they have been providing first aid education and organizing a first aid competition in the school every year).

Like the intervention school, the control school considers health promotion as part of environmental education.

### 2.3. Comparison of Health Promotion and Health Education Activities between the Intervention and Control Schools

Hungarian education legislation sets the same requirements for all schools in terms of health promotion. The most significant difference between the intervention and the control school in our study is that the intervention school has been operating a structured programme for a longer period of time within the framework of the Eco-School Programme, even before the regulation of the education system, they incorporated health promotion into their programme with a specific curriculum and tools (health promotion workbook), with teachers trained in health promotion and with the assistance of an external health education curriculum expert. The following table summarizes the differences and similarities between the two schools that are relevant to our study (Table 2).

### 2.4. Data Collection to Measure and Evaluate the Effectiveness of the Intervention

We collected data on health behaviour and well-being with an anonymous, self-administered paper-based questionnaire in a cross-sectional manner in both schools.

The health behaviour questionnaire was based on the Hungarian version of the Health Behaviour in School-Aged Children 2014 (HBSC 2014) survey [44] and the Hungarian European School Survey Project on Alcohol and Other Drugs 2015 (ESPAD 2015) [45].

#### 2.4.1. Sociodemographic Measures

The respondents’ sex, self-perceived family wealth, and parents’ education and employment status were measured. The subjective perception of family wealth was assessed on a five-point Likert scale ranging from “very well of” to “not at all well of”. The parents’ highest level of education was categorized as vocational school or less, secondary school or high school, university or college degree, and not known. The employment status categories were as follows: both parents were employed (active), and one of the parents was active or unknown [44,46].

#### 2.4.2. Measurement of Health Behaviour and Well-Being

Pupils were asked about their breakfast habits on weekdays and weekends, and responses were categorized as either having breakfast five times or less on weekdays and having breakfast two times or less on weekends [44,46].

The frequency of consumption of healthy (fruits, vegetables) and unhealthy (sweets, sugar-sweetened drinks, salty snacks, fast foods, energy drinks, and coffee) food was assessed with a seven-item Likert scale from “never” to “more than once a day”. To assess the intervention’s effectiveness, we combined categories to indicate favourable eating patterns [44,46]. We differentiated between daily and less-than-daily consumption of healthy and between less-than-weekly and at least weekly consumption of unhealthy food consumption. We considered individuals who never consumed coffee or energy drinks to be abstinent. To determine healthy and unhealthy eating, we used scales based on the frequencies of consumed vegetables, fruits, sweets, sugary soft drinks, energy drinks, salty snacks, and fast food [44,46]. Two scales were created from the variables: “healthy eating scale” and “unhealthy eating scale.” The scales were constructed using the same methodology as in previous publications [47,48,49]. First, the responses were converted into numerical values as follows: never = 0, less than once a week = 0.25, once a week = 1, 2–4 days a week = 3, 5–6 days a week = 5.5, and at least once a day = 7. In the second step, we summed the scores. The “healthy eating” scale is based on the sum of the scores for fruit and vegetable consumption, ranging from 0 to 14. We summed the scores for sweets, sugary soft drinks, energy drinks, salty crisps, and fast food consumption to obtain an “unhealthy eating” scale ranging from 0 to 35. A higher score on the scale indicates more frequent consumption of healthy or unhealthy foods. The scale was treated as a binary variable based on the 66th percentile (healthy diet) and the 33rd percentile (unhealthy diet) cut-off values of the scales, representing favourable outcomes on both scales.

The respondents’ moderate-to-vigorous physical activity (MVPA) and vigorous physical activity (VPA) were measured based on the HBSC 2014 methodology [44,50]. For analysis, the MVPA variable was dichotomized based on the cut-off point exercise performed at least 5 days per week [51]; while the threshold was being active at least twice weekly in the case of the VPA question [44,50].

Screen time was defined separately for weekdays and weekends, encompassing activities such as watching TV, videos, or DVDs; playing games on a computer or game console; and using a computer for email, the internet, or homework [44,50]. During the analyses, a favourable attitude was considered as using a maximum of 1 h daily [44].

Regarding smoking habits, we analysed the lifetime prevalence and frequency of regular cigarettes, electronic cigarettes (e-cigarettes), and hookah use in the last 30 days [44,45]. To analyse the effectiveness of our intervention, we dichotomized smoking variables considering nonsmokers as those who had not used any tobacco products [44,52].

Our study also recorded the lifetime prevalence and monthly occurrence of alcohol consumption, drunkenness, and binge drinking. Binge drinking was defined as the consumption of 5 or more units of alcohol in a single occasion, where a unit of alcohol means 250 mL of beer, 100 mL of wine, 60 mL of vermouth/liqueur, or 30 mL of a short drink [44,45]. As part of the analysis, we looked at the frequency of the abstinent category.

The pupils’ life satisfaction was measured by the Cantril ladder [44], where a score of 0 represents the worst possible life and 10 is the best possible life. We dichotomized this variable based on the HBSC 2014 methodology to analyse the effectiveness of the intervention. One group consisted of pupils who rated their life satisfaction as average or below (0–9 points). The other group was composed of pupils who rated their life satisfaction as 10 (above average).

A dichotomous variable was created for pupils’ self-rated health [44], where those who rated their health as excellent or good were grouped into one category, while the other category was composed of those who rated their health as fair, poor, or very poor.

### 2.5. Data Processing and Statistical Analyses

Data were collected among the 5th and 7th-grade pupils in the spring of 2017, 2018, 2019, and 2021. In 2020, we could not carry out the data collection due to the COVID-19 pandemic. Descriptive univariate analyses were used to assess the baseline characteristics of the participants. Differences between the intervention and control schools by grade were quantified using the χ^2^ test.

The effect of the intervention was quantified using univariate and multivariate logistic regression analyses that compared the data from the intervention and control schools stratified by grade. Univariate models included only schools as independent variables to show the crude intervention effects, while multivariate models were used to show the effect of the interventions on the studied outcome indicators independently of pupils’ sociodemographic factors such as sex, self-perceived family wealth, and parents’ education and employment status. These models were run on a pooled database containing data for all years, separately for the 5th and 7th grades, to investigate the potential effect of the intervention. The classical pre–post comparison did not seem to be feasible considering the ongoing nature of the health promotion programme in the intervention school. We described the relationships between dependent and independent variables using odds ratios (OR) and their associated 95% confidence intervals (95% CI). We employed the IBM SPSS 28.0.1.0 software package for the analyses.

## 3. Results

### 3.1. Basic Characteristics of the Participants

**Figure 1:**
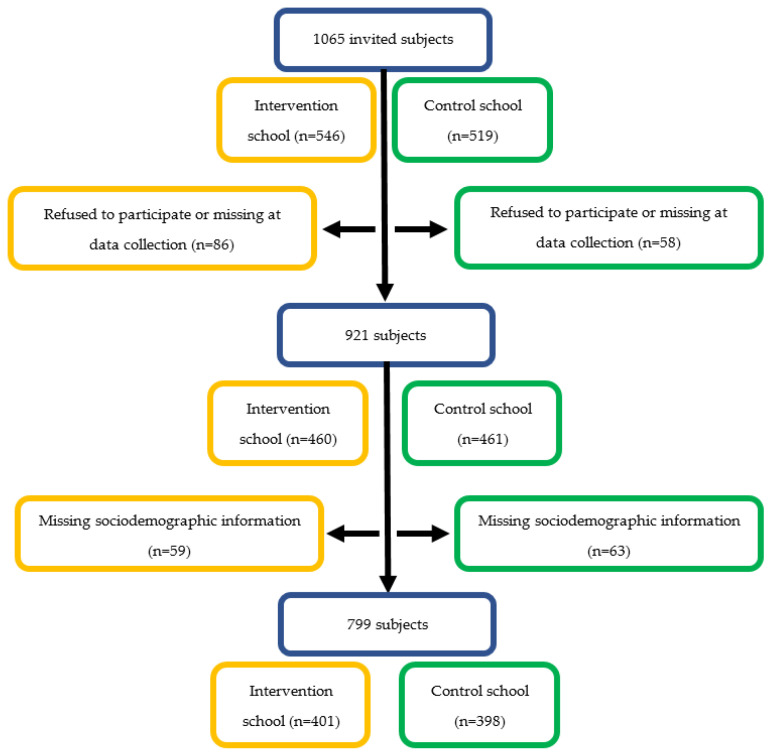
Flow diagram for studied participants included in the final analysis.

A total of 1065 pupils were invited to participate in the study. One hundred and twenty-seven pupils refused to participate in the study or were missing when the questionnaire was completed. In addition, three pupils’ data were excluded from the data analysis because they completed the questionnaire inconsistently. Altogether, only participants who provided complete information on sociodemographic variables were included in the analyses, resulting in a database with 799 records and an overall response rate of 75.02% (Figure 1).

In the intervention school, 54% of the 5th-grade pupils were male, compared to 47% in the control school. Among 7th-grade pupils, similar frequencies were observed for boys in the intervention school (51%) and in the control school (46%). In both the intervention and control schools and across all the studied school years, the majority of respondents reported having parents who were employed. Approximately 24% of the fathers of 5th-grade adolescents in the intervention school were skilled with a university or college degree, compared to 25% in the control school. Among the 7th-grade respondents, there was a significant deviation in fathers’ education attainment: in the intervention school, 25% of fathers had a university or college degree, compared to 20% in the control school. In all studied groups and grades, the frequency of mothers with a university or college degree was greater than that of fathers. Among 5th-grade pupils, there was a significant difference regarding subjective perception of family wealth: approximately 36% of the intervention school families and 48% of the control school families belonged to the very wealthy category. Among the respondents in the 7th grade, the proportion of very wealthy families was 19% in the intervention school and 17% in the control school (Table 3).

### 3.2. The Effectiveness of the Intervention

According to the univariate and multivariate models, the odds of daily fruit consumption were lower in the intervention group than in the control group among 5th-grade pupils. According to both the univariate and multivariate analyses, the intervention increased the odds of a lower unhealthy eating score among 5th-grade adolescents. We have found a similar effect in the case of the 7th-grade pupils during the multivariate analyses. (Table 4).

Among 5th graders who received the intervention, less screen time significantly increased (a maximum of 1 h of daily computer use on weekdays or weekends). Additionally, in the intervention group of 7th-grade pupils, the odds of fewer video games playing on weekdays (maximum of 1 h) were significantly lower than those in the control group. (Table 5).

The odds of lifetime smoking abstinence regarding cigarette, e-cigarette, and hookah use increased in the intervention group among the 7th-grade pupils. The intervention increased the odds of smoking abstinence regarding e-cigarette and hookah use in the past 30 days among 7th-grade pupils. (Table 6).

Among the 7th-grade pupils, both univariate and multivariate models suggested better self-perceived health in the intervention group compared to the control group. (Table 7).

## 4. Discussion

Our research team examined the effectiveness of the “Development of a Healthy Lifestyle in the Eco-School” primary school health promotion programme on pupils’ health behaviour and well-being from 2017 to 2021. We found a positive relationship between the programme and pupils’ health behaviour and well-being in some cases.

In terms of eating habits, we found significantly lower odds of daily fruit consumption among 5th-grade pupils in the intervention group. The difference was no longer noticeable among the 7th graders. At younger ages, the family’s eating habits and welfare can have a greater influence on children’s eating habits. The above difference may be due to a fundamental difference in family habits that the intervention has not been able to influence sufficiently [7,53,54]. This also seems likely because the difference disappears in the older age group in the 7th grade when adolescents are already trying to make their own decisions about their habits.

We found a significant association with the overall unhealthy eating scale score. Those who participated in the health promotion programme were more likely to avoid eating unhealthy foods than those in the control group. This may be because adolescents have more control over the consumption of unhealthy foods (e.g., what they spend their pocket money on), and family background is less important than the consumption of healthy foods. Thus, an intervention focusing on children may have had a greater impact on the consumption of unhealthy foods. In a previous study among adolescents, we found similar results [49].

The intervention did not show a relationship with pupils’ physical activity habits. One reason for this may be that the two schools provided similar opportunities for pupils to participate in different physical activity programmes and sports activities. The intervention school provided pupils with additional knowledge about physical activity, but as previous studies have shown [55,56,57], additional knowledge is necessary but not sufficient to shape the right behaviour (e.g., the phenomenon of cognitive dissonance). In addition, the physical activity habits of pupils may also be more deeply influenced by family habits and family affluence [7,58,59,60,61].

In terms of sedentary behaviour, in the univariate analyses, we found that among those seven-grade pupils who participated in the intervention, the odds of spending less time playing video games were significantly lower than those of the control group. This difference was no longer present in the multivariate model, which suggests that it cannot be attributed to the effect of the intervention but to other factors that we adjusted for in the analysis [62,63,64]. Children’s screen time can also be affected by their parents’ use of electronic devices [65].

We found that fifthgraders who received the intervention were significantly more likely to spend less time on email, the internet, or homework. This difference no longer exists among 7th graders, which might be because they are more likely to use computers for school-related tasks. While the health promotion programme addressed the useful and healthy use of leisure time, it did not promote a more conscious use of digital tools, partly because they were not widely used at the start of the programme. Probably this could be the reason why there was no major difference between the intervention and control groups in terms of sedentary screen time.

Smoking was less prevalent among 5th graders, so the association between tobacco product use and the intervention could not be examined. The intervention had a positive effect on smoking habits among 7th graders: the intervention group was more likely to have never tried cigarettes and hookah in their lifetime, and the odds of not using e-cigarettes and hookah in the past 30 days were also greater.

No significant association was found between alcohol consumption and the health promotion programme. However, for the odds ratios, there was a trend towards a positive effect on alcohol consumption (lifetime and last 30 days prevalence) and avoidance of drunkenness.

There was no significant association between the intervention and the pupils’ life satisfaction. Adolescents’ life satisfaction is more influenced by family structure, family support, and family welfare, which are not affected by the intervention [66,67].

The intervention had a positive impact on self-rated health among 7th graders. This may mean that through the knowledge and skills acquired during the intervention, pupils can have greater control over their well-being.

### 4.1. Strengths and Limitations

#### 4.1.1. Intervention

One of the limitations of the intervention was that due to the COVID-19 pandemic, schools had to choose an online teaching mode for several weeks from spring 2020 to spring 2021. During these periods, health promotion activities in school were either not carried out at all or were difficult to carry out. This may have affected our results. Furthermore, the intervention school involved the parents only in the organisation and implementation of the programme elements, and no special emphasis was placed on the education, skill development, or behaviour change of the parents during the programme.

The strength of the intervention is that it combines health promotion with environmental education, giving pupils a more complex understanding of what can affect their health. Moreover, the health promotion programme was based on a well-developed methodology, covered several themes, and ran over several years, involving teachers, parents, and external experts across the school. The programme is in line with the International Union for Health Promotion and Education (IUHPE) [16] criteria and is aligned with the NCC [35], which has been renewed in the meantime, and with the HHP [34,36] concept published in 2012.

#### 4.1.2. Evaluation

The certainty of our results could have been further increased if the intervention and control groups had also been premeasured for health behaviour and well-being, as some variance may have been masked by the potential baseline difference between the conditions of the pupils in the intervention and control schools.

A further limitation is that the effect of the intervention was measured using single-item questions. While such questions are a valid and widely used approach in epidemiological studies to assess the outcomes being studied, it is important to recognise that this approach may not fully capture the health behaviours of the pupils.

For statistical reasons, questionnaires with missing sociodemographic data were excluded from the data analysis, leading to a lower participation rate.

Every year during the study period, we sent feedback to the schools on the health behaviour of the pupils in relation to the national results but did not compare the two schools. We cannot rule out the possibility that, based on the feedback received, the control school may have focused more on health promotion alongside the intervention school. Although we have tried to map the health promotion activities taking place in the control school (see the methodology), knowing their results could indirectly lead to a greater focus on health promotion and influence teachers’ attitudes, which could minimally bias the results.

Last but not least, the cross-sectional nature of the study means that causality cannot be established and the long-term positive effects of the intervention are not known.

## 5. Conclusions

Improving the health of children and adolescents in schools is recommended since the school is the second largest socialization agent for young people after the family, and a well-developed school health promotion programme is a cost-effective solution for improving the health of the population. In the present study, we examined the effectiveness of a school health promotion programme that has been running for a longer period of time. The programme started before the Hungarian education system was regulated. It was implemented in a structured way, with a specific curriculum and tools, involving teachers trained in health promotion and an external health education curriculum expert.

Our results show that for those behaviours where pupils’ personal choices had a greater influence (unhealthy eating, smoking), the health promotion programme was more effective. For those behaviours where family background and parental influence were more pronounced (healthy eating, physical activity [7,53,54,58,59,60,61], screen time), the intervention had less impact. Self-perceived health was also favourable in the 7th-grade intervention group. Based on the above, by complementing the health promotion activities in the Hungarian school system with the elements used in the health promotion programme (teachers training in health promotion, health promotion lessons with a well-structured curriculum based on authentic workbooks, other health promotion activities through school and city programmes, involving parents and external experts), effective results can be achieved in terms of the health behaviour of pupils.

Our findings are in line with the conclusion of systematic reviews that more intensive, longer-term, multi-behavioural school health promotion programmes can be effective in promoting positive behaviour [18,19,20,21]. However, the presented health promotion programme should be further developed to increase its effectiveness. For instance, the workbooks and teaching materials used in the programme need to be improved and updated to meet the needs and challenges of the youth of today, e.g., digital health should be given greater emphasis. Furthermore, it would also be worthwhile to involve parents and several local organisations to a greater extent. This means, for example, increasing parents’ knowledge and skills, shaping attitudes, and working more closely with the local health promotion institute. In addition, the effectiveness of school health promotion programmes could increase if teachers have more opportunities to acquire knowledge, skills, and attitudes related to health promotion not only during in-service training but also during their university studies, as we concluded in our previous study [49].

All of the above would help to engage adolescents more often and more intensively with a preventive approach to life management.

## Figures and Tables

**Table 1:** Content of the health promotion workbook modules.

I. Healthy Lifestyle Module	II. Family and Social Life Module	III. Development of Self-Knowledge Module
- healthy eating - physical activity, health promoting exercises (e.g., eye and breathing exercises, stretching, relaxation) - personal and environmental hygiene - disease prevention - accident prevention, first aid - leisure activities - health services - environmental protection and sustainability	- developing social relationships - roles, role conflicts, conflict resolution - emotional literacy skills improvement - value formation, moral education - sexual maturation, sexual behaviour, friendship, love, matchmaking (from 6 to 8 grades)	- healthy self-image (self-esteem, self-confidence) - acceptance of difference - positive decision-making - conflict management, problem solving strategies - avoiding risk-taking behaviours and harmful addictions, strengthening good decision-making and the ability to say “no” - stress management and relaxation techniques

**Table 2:** School activities, programmes, and projects that may have had an impact on pupils’ health behaviour and attitudes towards health and well-being.

Intervention School	Both Schools	Control School
Eco-school from 2007.	**Eco-school** Both schools consider the physical, mental and social improvement of pupils as part of environmental education.	Eco-school from 2016.
Model health promotion programme launched in 2008: - organized by an external health education curriculum expert - structured health promotion lessons (18 lessons per school year), taught by qualified teachers for health promotion, based on an authentic workbook	**Health promotion programme**	Did not have a special health promotion programme like the intervention school.
- The topics and teaching-learning methods of the health promotion subject launched in 2008 were well integrated into the school curriculum and they are in line with the 2012 legislation and the objectives and requirements of the National Core Curriculum. - Health promotion in the classroom, as required by the NCC, was based on the topics and content of the workbooks used since 2008.	**Decree No. 20/2012 on Holistic Health Promotion** - Daily physical education - Holistic Health Promotion in schools - More comprehensive health promotion programme in the schools’ pedagogical programmes **National Core Curriculum (NCC) 2012 change** - Health education integrated into subjects.	
- Organizing several theme weeks and days on healthy lifestyles each year, with parental support - Health and sports days are organized several times a school year. - Pupils were regularly involved in the maintenance of the schoolyard (e.g., planting shrubs), learning about medicinal plants in the herb garden and composting.	**Theme weeks, health days, regular programmes** - Sustainability theme weeks 2016–2020 about topics related to environment and health with parental support - The school offers the opportunity to take part in a variety of sports and physical activities, so pupils can try out different sports. - Regular participation in interschool sports competitions.	- Once a school year, a healthy lifestyle competition is organized between teams of classes (1 day) - Participation in a running race organized by a charity once a year - Ice Skating Gala in the Debrecen Ice Hall, 1 day per school year (ice skating is part of the local curriculum). - A health and sport week is organized every Olympic year (once every 4 years) - Since the school year 2009/2010 they are partners of the Hungarian Red Cross, since then they have been providing first aid education and organizing a first aid competition in the school every year. - Organizing school dental competitions between 2010–2018
Other programmes on smoking (2 days) and cancer prevention (1 series of competitions) organized occasionally and/or involving only some of the pupils between 2010 and 2019.	**Other programmes** Participation with parents in city activities, nature walks, bike rides, running races, and lectures.	Other programmes on sport (2 days), gardening (2 days) and health (1 day) organized occasionally and/or involving only some of the pupils between 2010 and 2019.
Lessons on healthy lifestyles taught by nurses and school doctors, a few times a year in each class.		
The health promotion activities were cancelled in 2020–2021, when online education was provided as a result of COVID-19 pandemic.		

**Table 3:** Characteristics of the respondents who were included in the analyses and the differences between 5th and 7th-grade pupils at the intervention and control schools evaluated by the χ^2^ test.

		5th Grade			7th Grade		
		Intervention School	Control School	*p*-Value	Intervention School	Control School	*p*-Value
Sex	boy	101 (54.01%)	98 (47.34%)	0.186	109 (50.93%)	87 (45.55%)	0.279
	girl	86 (45.99%)	109 (52.66%)		105 (49.07%)	104 (54.45%)	
Employment status of the parents	not both parents active/not known	28 (14.97%)	26 (12.56%)	0.487	24 (11.21%)	25 (13.09%)	0.564
	both parents active	159 (85.03%)	181 (87.44%)		190 (88.79%)	166 (86.91%)	
Father’s education level	vocational school or less	35 (18.72%)	46 (22.22%)	0.511	48 (22.43%)	71 (37.17%)	**0.013**
	secondary school/high school	35 (18.72%)	42 (20.29%)		65 (30.37%)	45 (23.56%)	
	university or college degree	44 (23.53%)	53 (25.60%)		54 (25.23%)	38 (19.90%)	
	not known	73 (39.04%)	66 (31.88%)		47 (21.96%)	37 (19.37%)	
Mother’s education level	vocational school or less	33 (17.65%)	28 (13.53%)	0.711	36 (16.82%)	45 (23.56%)	0.369
	secondary school/high school	39 (20.86%)	47 (22.71%)		83 (38.79%)	66 (34.55%)	
	university or college degree	59 (31.55%)	70 (33.82%)		58 (27.10%)	46 (24.08%)	
	not known	56 (29.95%)	62 (29.95%)		37 (17.29%)	34 (17.80%)	
Subjective perception of family wealth	very wealthy	67 (35.83%)	100 (48.31%)	**0.003**	41 (19.16%)	33 (17.28%)	0.079
	quite wealthy	70 (37.43%)	46 (22.22%)		77 (35.98%)	50 (26.18%)	
	average or worse	50 (26.74%)	61 (29.47%)		96 (44.86%)	108 (56.54%)	
TOTAL		187 (100%)	207 (100%)		214 (100%)	191 (100%)	

Significant results are highlighted in bold.

**Table 4:** Impact of the intervention on dietary habits among 5th and 7th-grade pupils according to univariate and multivariate logistic regression models.

	Univariate Model		Multivariate Model *	
	5th Grade OR (95% CI)	7th Grade OR (95% CI)	5th Grade OR (95% CI)	7th Grade OR (95% CI)
Breakfast on every schooldays (ref.: less than 5 times)	1.01 (0.68; 1.51)	1.17 (0.79; 1.73)	1.07 (0.70; 1.62)	1.12 (0.75; 1.69)
Breakfast on every weekends (ref.: less than 2 times)	0.86 (0.48; 1.55)	1.03 (0.65; 1.65)	0.86 (0.46; 1.60)	1.02 (0.63; 1.65)
Daily fruit consumption (ref.: less than every day)	**0.56 (0.36; 0.86)**	0.73 (0.47; 1.13)	**0.58 (0.37; 0.92)**	0.67 (0.42; 1.07)
Daily vegetable consumption (ref.: less than every day)	0.86 (0.56; 1.33)	0.88 (0.57; 1.36)	0.94 (0.59; 1.48)	0.84 (0.53; 1.34)
Sweet consumption less than weekly (ref.: once or more a week)	1.41 (0.86; 2.32)	0.93 (0.56; 1.56)	1.38 (0.83; 2.32)	0.97 (0.57; 1.64)
Sugar sweetened drinks consumption less than weekly (ref.: once or more a week)	0.93 (0.61; 1.42)	1.31 (0.87; 1.97)	1.05 (0.67; 1.63)	1.37 (0.89; 2.11)
Snack consumption less than weekly (ref.: once or more a week)	1.10 (0.72; 1.69)	1.13 (0.75; 1.71)	1.17 (0.75; 1.82)	1.21 (0.79; 1.86)
Fast food consumption less than weekly (ref.: once or more a week)	1.17 (0.78; 1.77)	1.44 (0.95; 2.19)	1.10 (0.72; 1.70)	1.54 (0.99; 2.38)
Have not consume energy drinks (ref.: have consumed at least once in lifetime)	1.25 (0.73; 2.16)	1.12 (0.75; 1.69)	1.29 (0.73; 2.27)	1.16 (0.76; 1.76)
Have not consume coffee (ref.: have consumed at least once in lifetime)	1.06 (0.66; 1.71)	0.88 (0.59; 1.31)	1.10 (0.67; 1.80)	0.80 (0.53; 1.22)
Healthy eating (ref.: 66.66th percentile or below)	0.74 (0.49; 1.10)	0.78 (0.52; 1.17)	0.77 (0.51; 1.18)	0.76 (0.50; 1.16)
Unhealthy eating (ref.: 33.33rd percentile or above)	**1.56 (1.01; 2.39)**	1.53 (0.99; 2.34)	**1.72 (1.10; 2.71)**	**1.61 (1.03; 2.51)**

OR (95% CI): odds ratios (95% confidence intervals), ref.: reference category. * controlled for sex, subjective perception of family wealth, and the education and employment status of the parents; Significant differences are highlighted in bold.

**Table 5:** Impact of the intervention on physical activity and sedentary lifestyle among 5th and 7th-grade pupils according to univariate and multivariate logistic regression models.

	Univariate Model		Multivariate Model *	
	5th Grade OR (95% CI)	7th Grade OR (95% CI)	5th Grade OR (95% CI)	7th Grade OR (95% CI)
minimum 1 h MVPA 5 or more days a week (ref.: less than 5 days)	1.09 (0.73; 1.64)	0.92 (0.61; 1.38)	1.08 (0.71; 1.66)	0.88 (0.57; 1.35)
minimum 2 days VPA a week (ref.: less than 2 days)	1.06 (0.68; 1.66)	0.92 (0.60; 1.40)	1.11 (0.69; 1.77)	0.89 (0.57; 1.39)
maximum 1 h daily video, TV, DVD watching on weekdays (ref.: more than 1 h)	1.22 (0.82; 1.83)	0.98 (0.65; 1.48)	1.30 (0.85; 1.97)	0.99 (0.65; 1.53)
maximum 1 h daily video, TV, DVD watching on weekends (ref.: more than 1 h)	1.16 (0.70; 1.92)	1.06 (0.61; 1.85)	1.25 (0.74; 2.09)	1.13 (0.63; 2.02)
maximum 1 h daily computer or game console playing on weekdays (ref.: more than 1 h)	1.09 (0.72; 1.64)	**0.67 (0.45; 0.99)**	1.26 (0.81; 1.96)	0.70 (0.45; 1.08)
maximum 1 h daily computer or game console playing on weekends (ref.: more than 1 h)	0.87 (0.56; 1.37)	0.70 (0.46; 1.07)	0.95 (0.58; 1.54)	0.76 (0.47; 1.22)
maximum 1 h daily for email, internet or homework on weekdays (ref.: more than 1 h)	**1.53 (1.01; 2.31)**	0.87 (0.59; 1.30)	**1.60 (1.03; 2.47)**	0.91 (0.60; 1.39)
maximum 1 h daily for email, internet or homework on weekends (ref.: more than 1 h)	**1.89 (1.24; 2.89)**	1.02 (0.66; 1.58)	**2.00 (1.28; 3.11)**	1.16 (0.73; 1.84)

MVPA. moderate-to-vigorous physical activity, VPA: vigorous physical activity, OR (95% CI): odds ratio (95% confidence interval), * controlled for sex, subjective perception of family wealth, and the education and employment status of the parents, Significant differences are highlighted in bold.

**Table 6:** Impact of the intervention on smoking and alcohol consumption among 5th and 7th-grade pupils according to univariate and multivariate logistic regression models.

	Univariate Model		Multivariate Model *	
	5th Grade OR (95% CI)	7th Grade OR (95% CI)	5th Grade OR (95% CI)	7th Grade OR (95% CI)
Have not used cigarettes (ref.: have used it at least once in their lifetime)	1.82 (0.45; 7.38)	2.11 (1.04; 4.29)	1.89 (0.43; 8.19)	2.21 (1.06; 4.64)
Have not used cigarettes in the past 30 days (ref.: have used it at least once in the last 30 days)	-	2.00 (0.58; 6.95)	-	1.85 (0.47; 7.24)
Have not used e-cigarettes (ref.: have used it at least once in their lifetime)	1.44 (0.55; 3.80)	1.68 (0.95; 2.99)	1.57 (0.57; 4.33)	1.84 (1.00; 3.36)
Have not used e-cigarettes in the past 30 days (ref.: have used it at least once in the last 30 days)	1.38 (0.38; 4.96)	2.33 (0.92; 5.90)	1.42 (0.37; 5.47)	**2.76 (1.05; 7.24)**
Have not used hookah (ref.: have used it at least once in their lifetime)	1.85 (0.62; 5.50)	1.65 (0.90; 3.00)	1.89 (0.61; 5.90)	**1.88 (1.01; 3.52)**
Have not used hookah in the past 30 days (ref.: have used it at least once in the last 30 days)	-	2.59 (0.88; 7.58)	-	**4.09 (1.26; 13.28)**
Alcohol abstinent (ref.: have consumed it at least once in their lifetime)	0.77 (0.46; 1.31)	1.31 (0.88; 1.94)	0.79 (0.45; 1.38)	1.31 (0.87; 1.97)
Alcohol abstinent for the last 30 days (ref.: have consumed it at least once in the last 30 days)	0.57 (0.18; 1.76)	1.01 (0.59; 1.74)	0.50 (0.15; 1.62)	1.04 (0.60; 1.81)
Avoidance of drunkenness (ref.: have been drunk at least once in their lifetime)	1.31 (0.41; 4.19)	1.32 (0.71; 2.45)	1.28 (0.38; 4.39)	1.46 (0.76; 2.79)
Avoidance of drunkenness for the last 30 days (ref.: have been drunk at least once in the last 30 days)	0.93 (0.06; 15.04)	1.34 (0.44; 4.05)	0.96 (0.05; 19.56)	1.45 (0.46; 4.55)
No binge drinking (ref.: binge drinking at least once in their lifetime)	0.70 (0.33; 1.50)	0.82 (0.48; 1.42)	0.72 (0.33; 1.60)	0.83 (0.47; 1.47)
No binge drinking for the last 30 days (ref.: binge drinking at least once in the last 30 days)	2.29 (0.44; 11.97)	0.54 (0.21; 1.38)	1.92 (0.35; 10.57)	0.53 (0.20; 1.40)

OR (95% CI): odds ratio (95% confidence interval); -: cannot be analysed due to low case numbers; * controlled for sex, subjective perception of family wealth, and the education and employment status of the parents. Significant differences are highlighted in bold.

**Table 7:** Impact of the intervention on life satisfaction and self-perceived health among 5th and 7th-grade pupils according to univariate and multivariate logistic regression models.

	Univariate Model		Multivariate Model *	
	5th Grade OR (95% CI)	7th Grade OR (95% CI)	5th Grade OR (95% CI)	7th Grade OR (95% CI)
Life satisfaction is above average (ref.: average or below)	1.16 (0.62; 2.16)	1.28 (0.77; 2.15)	1.33 (0.69; 2.57)	1.18 (0.69; 2.03)
Self-rated health is good or excellent (ref.: fair or poor)	1.35 (0.89; 2.05)	**2.02 (1.35; 3.01)**	1.47 (0.94; 2.83	**1.97 (1.29; 2.99)**

OR (95% CI): odds ratio (95% confidence interval); * controlled for sex, subjective perception of family wealth and the education and employment status of the parents. Significant differences are highlighted in bold.

## Data Availability

The raw data supporting the conclusions of this article will be made available by the authors upon request. The data are not publicly available due to the participants of this study did not give written consent for their data to be shared publicly.

## Appendix A

**Table A1:** Methods of the school health promotion programme to promote positive health behaviours among pupils in light of the COM-B model.

**Capability**
**Methods for shaping abilities and skills**
**Physical capacity:** Health promoting exercises in the workbook help improve physical and mental health, body control, and “more focused presence”.
**Psychological capacity:** Knowledge and skills development with well-structured curriculum and trained teachers in health promotion covering the following topics and areas: basic biological, anatomical, and physiological knowledge and skills development in three modules: healthy lifestyle, family and social life, development of self-knowledge; playful tasks, experimental tasks, situational practices, tasks to encourage monitoring, reflection and improvement of lifestyle and behaviour.
**Motivation**
**Methods used to foster motivation**
**Creating intention (reflective processes), attitude-forming:** Main message: Health is a value that needs to be protected and developed. Playful tasks, experimental tasks, situational practices, tasks to encourage monitoring, reflection and improvement of lifestyle and behaviour. Avoiding risk-taking behaviours and harmful addictions, strengthening good decision-making and the ability to say “no” to psychoactive substances. Take part in school, city and other organisations’ events and competitions (sport, cancer prevention, smoking prevention). Presence and encouragement of teachers trained in health promotion and health education curriculum expert.
**Habits (automatic processes):** **Strengthening and striving to learn good habits:** ○ Tasks to encourage monitoring, reflection and improvement of lifestyle and behaviour. ○ Positive decision-making. **Striving to stop unfavourable habits** ○ Positive decision-making. ○ Avoiding risk-taking behaviours and harmful addictions, strengthening good decision-making and the ability to say “no” to psychoactive substances.
**Opportunities**
**The environment that supports the intervention as a resource**
**Social environment:** School management, teachers who trained in health promotion. School community. Parents (who support or take part health promotion activities and who have been involved in the organisation and implementation of health promotion and environmental education programmes). The work of the local public health bodies
**Physical environment: School** Opportunity to sport events Creating a healthier school environment Maintenance of the schoolyard with the involvement of pupils, learning about herbs in herb garden care and composting experiencing the joy of gardening Schools and classroom decorations for the duration of theme days and theme weeks
